# Comparison of current relative value unit-based prices and utility between common surgical procedures, including orthopedic surgeries, in South Korea

**DOI:** 10.1186/s12962-024-00538-z

**Published:** 2024-04-11

**Authors:** Yoon Hyo Choi, Tae Hun Kwon, Chin Youb Chung, Naun Jeong, Kyoung Min Lee

**Affiliations:** Department of Orthopedic Surgery, Seoul National University Bundang Hospital, 300 Gumi-Dong, Bundang-Gu, Seongnam-Si, Gyeonggi South Korea

**Keywords:** Relative value unit, QALY, Medical fee schedule, CEA

## Abstract

**Background:**

The medical pricing system strongly influences physicians’ job satisfaction and patient health outcomes. This study aimed to investigate the current relative value unit (RVU)-based pricing and utility of patients in commonly performed surgical procedures in South Korea.

**Methods:**

Fifteen common surgical procedures were selected from OECD statistics, and three additional orthopedic procedures were examined. The current pricing of each surgical procedure was retrieved from the Korea National Health Insurance Service, and the corresponding utilities were obtained as quality-adjusted life year (QALY) gains from previous studies. The relationship between the current prices (RVUs) and the patients’ utility (incremental QALY gains/year) was analyzed. Subgroup analysis was performed between fatal and non-fatal procedures and between orthopedic and non-orthopedic procedures.

**Results:**

A significant negative correlation (r = − 0.558, p < 0.001) was observed between RVU and incremental QALY among all 18 procedures. The fatal subgroup had a significantly higher RVU than the non-fatal subgroup (p < 0.05), while the former had a significantly lower incremental QALY than the latter (p < 0.001). Orthopedic procedures showed higher incremental QALY values than non-orthopedic procedures, but they did not show higher prices (RVU).

**Conclusions:**

This paradoxical relationship between current prices and patient utility is attributed to the higher pricing of surgical procedures for fatal and urgent conditions. Orthopedic surgery has been found to be a cost-effective treatment strategy. These findings could contribute to a better understanding of the potential role of incremental QALY in pursuing value-based purchasing or reasonable modification of the current medical fee schedule.

## Background

In South Korea, annual surgical spending has been increasing, with expectations of further growth in the future. However, most surgeons lack awareness of the values or costs of medical or surgical care [1] Several researchers have argued that the current medical fee schedule is undervalued and arranged unequally across surgical specialties [2, 3].

Determining the cost of a specific surgical procedure is debatable and problematic. To assess surgeons as providers of medical services, the relative value units (RVUs) could be a feasible model reflecting their work intensity, time, and equipment investment [4], while the cost-effectiveness model could be used to assess patients as consumers of medical services that provide equivalent utility to the payment [5, 6] The quality-adjusted life year (QALY) has been a useful tool for evaluating the effectiveness of medical services, which is life expectancy adjusted for health-related quality of life [7].

In a traditional fee-for-service system, physician reimbursement is compensated regardless of the treatment outcome or patient satisfaction [8] To overcome this limitation, upcoming healthcare reform in South Korea seeks to transform the current medical fee schedule paradigm into a pay-for-performance or value-based purchasing (VBP) [9–11] Despite the emergence of VBP and its potential efficacy, the transition from fee-for-service to pay-for-performance has only made slow progress and encountered barriers due to the lack of consensus on which measurement represents the patient satisfaction the best [8, 12] Recently, with the increasing demand and interest in patient rights, cost-effectiveness analyses (CEA) based on QALY measurement have emerged as a possible answer to this challenge [5].

Therefore, this study aimed to investigate the disparities between provider-based RVU and consumer-based QALY for common surgical procedures. This information is expected to provide insights into the proper medical pricing and payment system for future adjustments. Utilizing accessible Korean data, our study seeks to address these crucial issues in the local healthcare context.

### Contribution/Significance of this article

- Investigating the disparities between provider-based relative value units (RVUs) and consumer-based quality-adjusted life years (QALYs) for common surgical procedures.
- Providing insights into the potential limitations of the current medical fee schedule paradigm and its implications for physician compensation and patient outcomes.
- Proposing a novel approach to comparing QALY values across diverse surgical specialties, offering a comprehensive analysis of the value of medical interventions.
- Offering valuable implications for policymakers, healthcare administrators, and practitioners in optimizing medical pricing systems to align with patient-centered care and value-based healthcare delivery, thereby enhancing overall healthcare quality and efficiency.

## Methods

### Data source and RVU

Fifteen common operations were quoted from the Organisation of Economic Co-operation and Development statistics, which offered information on healthcare across the 38 developed countries [13] Additionally, three common orthopedic surgeries available in CEA studies were included [14–16], which led to a final selection of 18 surgical procedures: cataract surgery, transluminal coronary angioplasty, coronary artery bypass graft (CABG), laparoscopic cholecystectomy, open cholecystectomy, repair of inguinal hernia, total hip replacement arthroplasty, total knee replacement arthroplasty, laparoscopic appendectomy, total laparoscopic hysterectomy, tonsillectomy and/or adenoidectomy, partial mastectomy, modified radical mastectomy, transurethral prostatectomy, extensive prostatectomy, ACL (anterior cruciate ligament) reconstruction, total shoulder arthroplasty, and lumbar spine fusion.

The RVUs for each surgical procedure were obtained from the Korea National Health Insurance Service (KNHIS) [17]. The mean relative value unit (RVU) values corresponding to each surgical procedure have been computed.

**Fig. 1:**
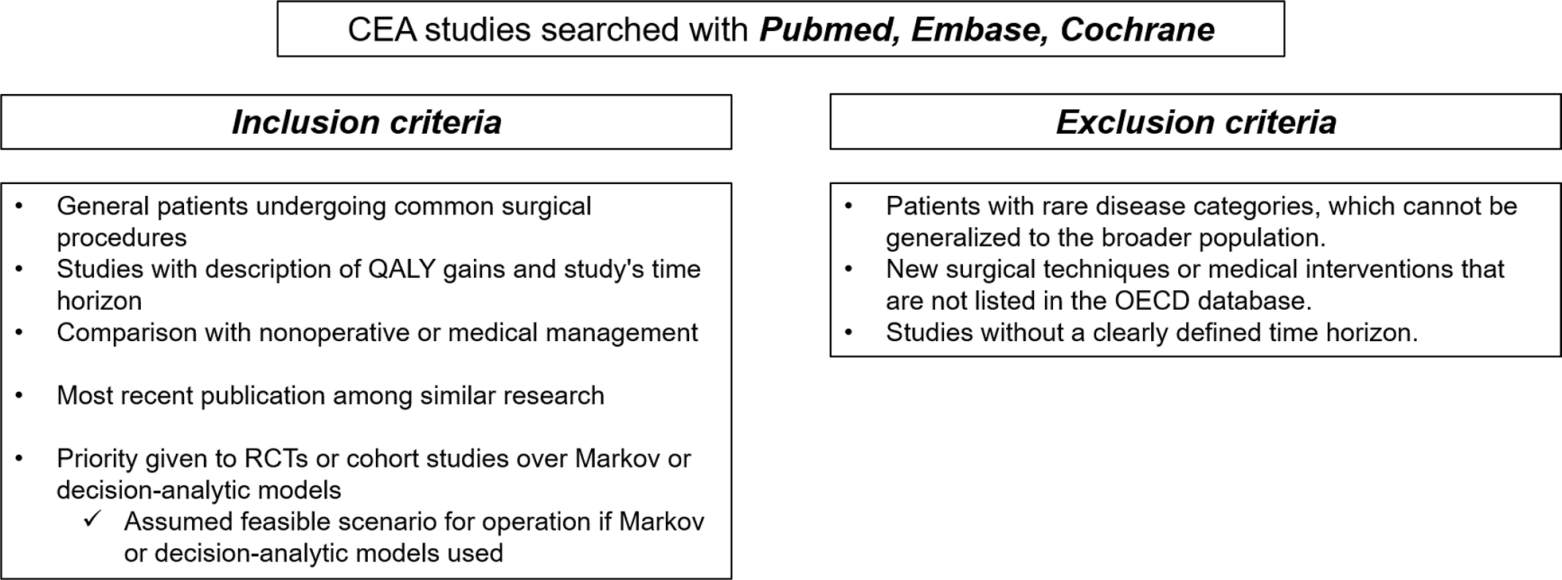
Criteria for the selection of CEA studies representative of common surgical procedures

The QALY values for each surgical procedure were extracted from cost-effectiveness analysis (CEA) studies meeting specific inclusion and exclusion criteria. These CEA studies were meticulously sourced through PubMed, Embase, and Cochrane databases, and subsequent selection followed stringent criteria. Inclusion criteria encompassed studies that: (1) involved general patients undergoing common surgical procedures aligned with their diagnosed conditions, (2) provided a comprehensive description of both QALY gains and the study's time horizon, (3) were the most recently published among similar research, (4) compared the specific surgery with nonoperative or medical management rather than other surgical options, and (5) favored randomized controlled trials (RCTs) or cohort studies over Markov or decision-analytic models when available. In cases where Markov or decision-analytic models were employed, we assumed the most realistic scenario for the operation, such as a common diagnosis and base case scenario. Exclusion criteria comprised studies that focused on: (1) patients with rare disease categories instead of common diagnoses, and (2) studies lacking a defined time horizon (Fig. 1).

### Calculation of incremental QALY

Traditionally, QALY gains have been used to compare the effectiveness of a specific procedure for the same diagnosis. It has generally been employed to evaluate the cost-effectiveness of interventions using an incremental cost-effectiveness ratio [15]. However, the principal disadvantage of using QALY gains is that it is limited to making a comparative study between different treatment modalities because of the disparity in the measuring period or time frame. Therefore, we designated the incremental QALY as a ΔQALYs/time frame to estimate and compare the effectiveness of an intervention per year for various surgical procedures [18].

### Definition of subgroups

Procedures were categorized into two subgroups: fatal and non-fatal. The fatal subgroup included transluminal coronary angioplasty, coronary artery bypass grafting, laparoscopic cholecystectomy, open cholecystectomy, laparoscopic appendectomy, total laparoscopic hysterectomy, partial mastectomy, modified radical mastectomy, and extensive prostatectomy. Non-fatal cases included cataract surgery, repair of inguinal hernia, total hip replacement arthroplasty, total knee replacement arthroplasty, ACL reconstruction, total shoulder arthroplasty, lumbar spine fusion, tonsillectomy and/or adenoidectomy, and transurethral prostatectomy.

Subgroup analysis was also performed between orthopedic surgical (OS) and non-OS procedures.

### Statistical analysis

For the statistical analysis, three variables were analyzed. Firstly, the nominal variable representing the name of each surgical procedure was considered. Secondly, the mean value of the RVU corresponding to each procedure was evaluated. Lastly, incremental QALY was examined.

Data normality was assessed using the Shapiro–Wilk test, and nonparametric data were compared between the two subgroups (e.g., fatal vs. non-fatal, or OS vs. non-OS) using the Mann–Whitney U-test. A scatter plot was constructed, and Spearman’s correlation analysis was performed to explore the potential correlation between RVU and incremental QALY.

All statistical analyses were conducted using SPSS software, version 26 (IBM Corp., NY, USA), and a two-sided p < 0.05 was considered significant.

## Results

The median value of RVU for all 18 procedures was 8715.39, and the interquartile range was 10,562.18. The CABG showed the highest RVU (36,932.31), followed by transluminal coronary angioplasty (19,572.6), while the tonsillectomy group had the lowest value (1075.21). Cataract surgery (3493.94) and laparoscopic appendectomy (3575.70) were evaluated below the first quartile (Table 1).

**Table 1:** Physician fee schedule and QALY gains of common operations

Operation	Mean RVU	QALY gains (Baseline QALY)	Time horizon (year)	Incremental QALY (QALY/year)	References	
					Author	Diagnosis (Study design)
Common operative procedures						
Cataract surgery	3493.94	2.52	14	0.18	Brown [19]	Cataract (Cohort)
Transluminal coronary angioplasty	19,572.6	0.26	5	0.05	Brandão [20]	Multivessel CAD (RCT)
Coronary artery bypass graft	36,932.31	0.23	5	0.05	Brandão [20]	Multivessel CAD (RCT)
Laparoscopic cholecystectomy	11,627.31	1.74	24	0.07	Sutherland [21]	Cholecystitis (Cohort)
Open cholecystectomy	25,518.26	0.35 (0.77)^a^	5	0.07	Mestral [22]	Cholecystitis (Markov)
Repair of inguinal hernia	6027.75	0.20	1	0.20	Palmqvist [23]	Inguinal hernia (Cohort)
Total Hip replacement arthroplasty	10,348.39	0.37	1	0.37	Rolfson [24]	Osteoarthritis (Cohort)
Total knee replacement arthroplasty	9118.73	1.33	5	0.27	Dakin [25]	Osteoarthritis (Cohort)
Laparoscopic appendectomy	3575.70	0.0773	1	0.08	Sceats [26]	Appendicitis (Markov ;20 year old)
Total Laparoscopic hysterectomy	8531.71	0.08 (0.82)^a^	1	0.08	Lundin [27]	Endometrial cancer (RCT)
Tonsillectomy and/or adenoidectomy	1075.21	0.33 (0.63)^a^	1	0.33	Bagwell [28]	Obstructive sleep apnea (Decision analytic)
Partial mastectomy	4498.70	0.923 (0.89)a	5	0.18	Polsky [29]	Breast cancer (Cohort)
Modified radical mastectomy	15,755.97	0.672 (0.89)^a^	5 (Survival 2.7 year)^b^	0.13	Polsky [29]	Breast cancer (Cohort)
Transurethral prostatectomy	7179.56	0.1	20	0.01	DiSantostefano [30]	BPH (Markov; 65y old)
Extensive prostatectomy	13,599.39	0.38	10	0.04	Sanghera [31]	Prostate cancer (Markov; > 65y old)
Other orthopedic procedures						
ACL reconstruction	5379.05	0.03	2	0.015	Lubowitz [16]	ACL rupture (Cohort)
Total shoulder arthroplasty	9560.55	1.02	2	0.51	Renfree [32]	Shoulder arthropathy (Cohort)
Lumbar spine fusion	8077.98	0.14	1	0.14	Passias [33]	Spondylolisthesis (Cohort)

*RVU* work relative value unit, *QALY* quality-adjusted life years. *CAD* Coronary artery disease. *BPH* Benign prostate hyperplasia. Baseline QALY was cited from

^a^Tengs [34] and the survival year of modified radical mastectomy was cited from

^b^Johnstone [35]

Incremental QALY (QALY/year) of all selected operations ranged from 0.01 to 0.51. The median value of the incremental QALY was 0.14, and the interquartile range was 0.13. Total shoulder arthroplasty yielded the highest QALY per year (0.51) compared to nonsurgical management, whereas TURP resulted in the lowest incremental QALY (0.01) compared to watchful waiting (Table 1).

**Fig. 2:**
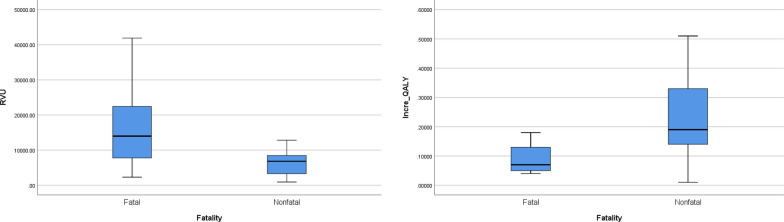
Comparison between the fatal and non-fatal subgroups. **A**: The fatal subgroup shows a higher RVU than the non-fatal group. **B**: The incremental QALY of the non-fatal subgroup is higher than that of the fatal subgroup

**Fig. 3:**
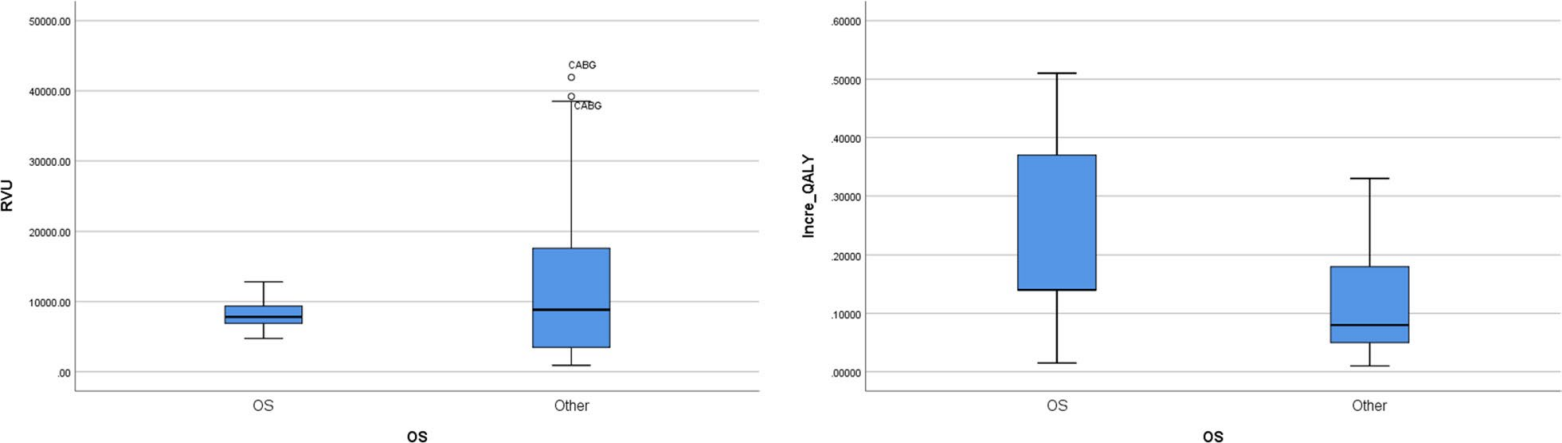
Comparison between the OS and non-OS procedures. **A**: OS procedures show similar RVU to those of non-OS procedures. **B:** Incremental QALYs of the OS procedures are higher than that of non-OS procedures

The fatal subgroup had a significantly higher RVU than the non-fatal subgroup (p < 0.05), while the former had a significantly lower incremental QALY than the latter (p < 0.001) (Fig. 2). The RVUs of OS procedures were not significantly different from that of non-OS procedures (p = 0.77), while incremental QALYs of OS procedures were significantly higher than non-OS procedures (p < 0.05) (Fig. 3).

**Fig. 4:**
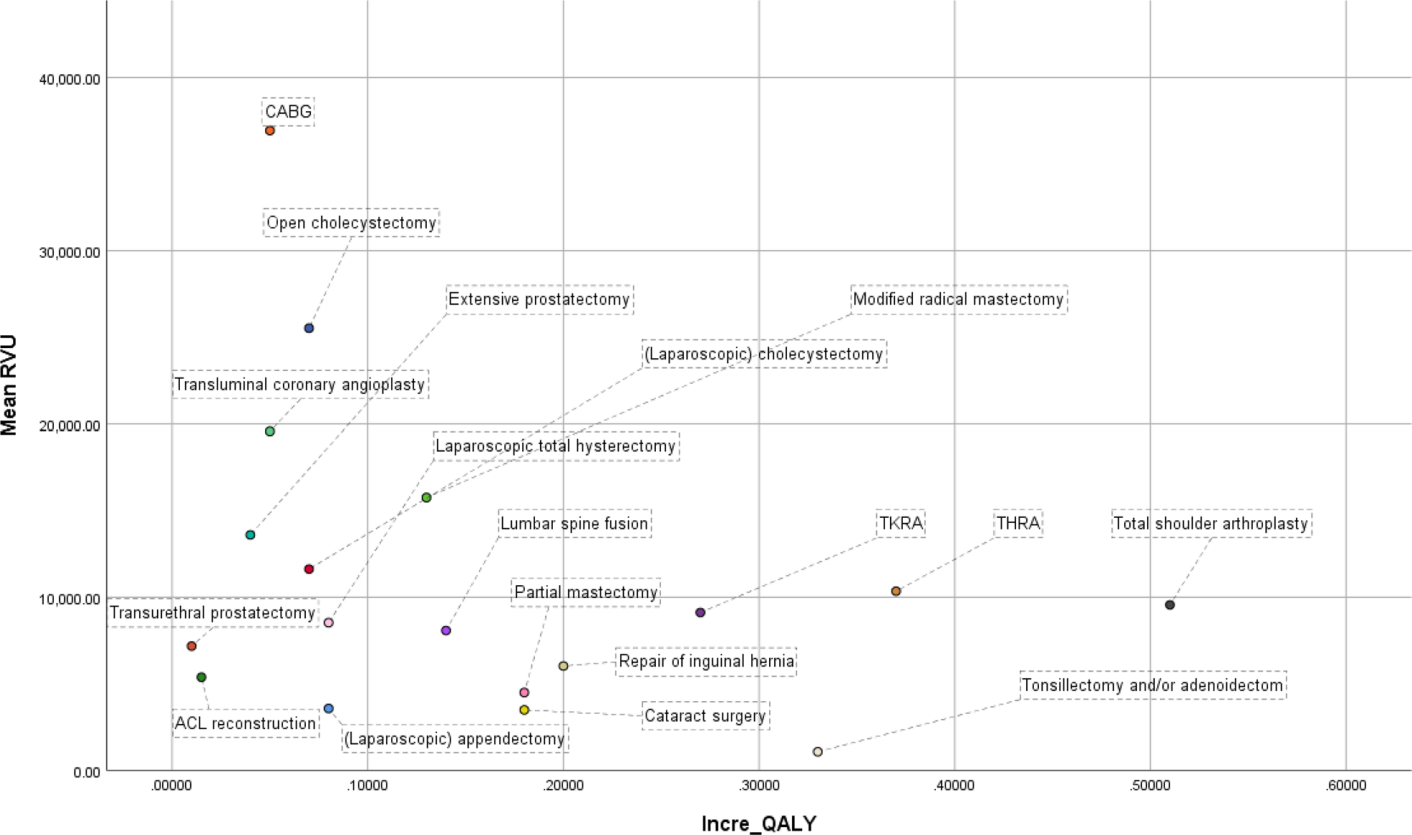
A scatter plot of mean RVUs and incremental QALYs of the 18 most common surgical procedures

There was a significant negative correlation between incremental QALY and RVU (r = − 0.558, p < 0.001) among all 18 procedures (Fig. 4). The correlation between the two was negatively significant (r = − 0.654, p < 0.001) in the fatal subgroup, while it was not significant (r = − 0.028, p = 0.887) in the non-fatal subgroup. There was a significant positive correlation between RVUs and QALYs among the OS procedures (r = 0.542, p < 0.05), while there was a significant negative correlation between the non-OS procedures (r = − 0.762, p < 0.001).

## Discussion

Determining the appropriate price for a specific procedure is a complex and debatable task for medical services. Conflicts could originate from the disparities between provider-based pricing and consumer-based benefits. In this study, we found a negative correlation between RVUs and incremental QALY in the most common surgical procedures in South Korea, which could be interpreted as a disparity between surgeon-based (provider-based) pricing and patients’ (consumers’) utility.

RVU is the basic component of the resource-based relative value scale (RBRVS), which is a methodology used by the Centers for Medicare and Medicaid services and private payers to determine physician payments [36] KNHIS adopted the RVU by employing the United States RBRVS system in 2001, where the payment was determined by the physician workload, practice expense, equipment cost value, and material cost value. Such provider-based pricing is the basis for physician compensation and reimbursement in the current healthcare insurance system. However, it has been affected by unrelated social factors such as political issues, which could lead to distorted and undervalued medical prices in South Korea. [37] In addition, RVU does not reflect the quality of the medical services or their values to patients, and some previous studies have criticized that the intensity, mostly estimated via a series of surveys or expert opinions from the Relative Value Scale Update Committee, did not reflect the valuation of surgical procedures pertinently [38–41].

CEA has drawn attention to the importance of overcoming the problems of the current medical pricing system [5]. This was an attempt to measure the appropriate price compared to the effectiveness or utility of a medical procedure, where effectiveness is most frequently evaluated by QALY gain. [7] QALY measures the number of life years adjusted by quality. Therefore, in an ideal medical pricing system, the QALY gain should be perfectly correlated with RVU [42] However, our study revealed a negative correlation between RVU and QALY gains, indicating a discrepancy in the current medical pricing system in South Korea. This discrepancy suggests that the current system places a higher value on procedures directly affecting patient fatality rather than those improving health-related quality of life. Furthermore, subgroup analysis within fatal procedures demonstrated that even among these, RVU and QALY exhibited a negative correlation. Specifically, more urgent procedures such as CABG, open cholecystectomy, and transluminal coronary angioplasty were associated with higher costs compared to less urgent ones like cancer surgeries including mastectomy, prostatectomy, and hysterectomy. These findings suggest that the current medical pricing system in South Korea prioritizes fatal and urgent surgical procedures, potentially at the expense of procedures with significant health-related quality of life improvements.

Although our study discriminated non-fatal from fatal procedures, non-fatal procedures such as total hip and knee replacement could possibly cause decreased mortality [43]. Indirect health benefits from increased mobility and activity levels following surgery may be a reason for this [44]. Therefore, we propose that non-fatal procedures should not be undervalued because they do not appear to have direct and apparent effects on the quantitative life gain. In addition, indirect social costs from osteoarthritis are not negligible, including missed workdays, income, employment, and disability payments [45]. In this context, common and representative orthopedic surgeries were found to be cost-effective in terms of the comparison between the price (RVU) and effectiveness (QALY gains).

While the satisfaction of surgeons in their profession is influenced by various factors beyond just income [46], appropriate pricing and compensation for specific surgical procedures are also reported to be crucial contributors to surgeons' job satisfaction, which is closely related to health outcomes [37]. The current RVU based pricing system does not sufficiently reflect patient utility in common surgical procedures in South Korea. VBP or a reasonable modification of the current medical fee schedule may be necessary, and because of the economic and social impact of medical costs in South Korea, determining the medical price needs to be based on social agreement.

This study has some limitations that must be considered when interpreting the results. First, the common operations quoted from OECD states may not fully represent the full spectrum of surgeries performed in each surgical specialty, and a selection bias might be present. Additionally, the small sample size may have limited our statistical power to nonparametric tests. Second, there is a possible mismatch between the actual surgical outcomes and QALY cited from CEA studies. Due to lack of evidence, a sensitivity analysis could not be performed. Third, in employing the notion of incremental QALY, we assumed time-utility independence, similar to the standard QALY model, that specific health utility persists as a constant value [47]. This assumption might overlook health utility, which generally declines over time and can vary depending on multiple factors, such as the age of disease onset, disability weight, and the duration of the disability [7]. Lastly, the reliance on data from cited papers may introduce potential limitations in terms of generalizability to the South Korean population.

Despite these limitations, our study possesses several strengths that enhance its contribution to understanding the disparities between provider-based RVUs and consumer-based QALYs for surgical procedures. Firstly, by conducting a comprehensive analysis encompassing various surgical interventions, we provide a holistic view of the challenges within medical pricing systems. Secondly, the incorporation of QALYs offers a patient-centered perspective, enriching the relevance and applicability of our findings in clinical practice. Lastly, our study lays a solid foundation for future research endeavors aimed at further elucidating the relationship between provider-based metrics and patient-centered outcomes, thus fostering ongoing advancements in healthcare delivery and pricing strategies.

In conclusion, this study demonstrated that the RVU and incremental QALY of common operations were disproportionate, with procedures associated with higher potential fatality being priced higher. Orthopedic surgeries, however, proved to be cost-effective in terms of patient utility. These findings could contribute to a better understanding of the potential role of incremental QALY in pursuing VBP or the reasonable modification of the current medical fee schedule, which should be based on social agreement as well as healthcare policy.

## Data Availability

The RVU datasets generated and/or analysed during the current study are available in the KNHIS repository, [https://www.data.go.kr/data/15067456/fileData.do]. And QALY datasets analysed during this study are included in the reference articles.

## Abbreviations

ACL: Anterior cruciate ligament
BPH: Benign prostate hyperplasia
CABG: Coronary artery bypass graft
CAD: Coronary artery disease
CEA: Cost-effectiveness analyses
KNHIS: Korea National Health Insurance Service
QALY: Quality-adjusted life year
RBRVS: Resource-based relative value scale
RVU: Relative value unit
VBP: Value-based purchasing
